# Verification of the Applicability of the FAD Method Based on Full-Scale Pressurised Tensile Tests of Large-Diameter X80 Pipelines

**DOI:** 10.3390/ma19030465

**Published:** 2026-01-23

**Authors:** Xiaoben Chen, Ying Zhen, Hongfeng Zheng, Haicheng Jin, Rui Hang, Xiaojiang Guo, Jian Xiao, Hao Zhou

**Affiliations:** 1 Construction Project Management Branch of National Petroleum and Natural Gas Pipeline Network Group Co., Ltd., Langfang 065000, China; 2 College of Storage & Transportation and Architectural Engineering, China University of Petroleum (Huadong), Dongying 257099, China; 3 China Petroleum Pipeline Research Institute Co., Ltd., Langfang 065000, China17629175034@163.com (H.Z.)

**Keywords:** girth weld defects, X80 pipeline, failure assessment diagram, applicability evaluation, stress-based approach

## Abstract

The Failure Assessment Diagram (FAD), as a significant method for evaluating the suitability of defective metallic structures, has been subject to considerable debate regarding its applicability in assessing ring welded joints for high-grade steel and large-diameter pipelines. To address this issue, this study first designed and conducted two sets of full-scale pressure-tension tests on large-diameter X80 pipeline ring welded joints, considering factors such as different welding processes, joint configurations, defect dimensions, and locations. Subsequently, three widely adopted failure assessment diagram methodologies—BS 7910, API 579, and API 1104—were selected. Corresponding assessment curves were established based on material performance parameters obtained from the ring weld tests. Finally, predictive outcomes from each assessment method were compared against experimental data to investigate the applicability of failure assessment diagrams for evaluating high-strength, large-diameter, thick-walled ring welds. The research findings indicate that, under the specific material and defect assessment conditions employed in this study, the API 1104 assessment results exhibited significant conservatism (two sets matched). Conversely, the BS 7910 and API 579 assessment results showed a high degree of agreement with the experimental data (eight sets matched), with the BS 7910 assessment providing a relatively higher safety margin compared to API 579. The data from this study provides valuable experimental reference for selecting assessment methods under specific conditions, such as similar materials, defects, and loading patterns.

## 1. Introduction

Currently, X80 high-strength steel has seen extensive application within the pipeline industry, becoming the dominant material for constructing high-pressure, large-diameter natural gas pipelines globally [1]. As the most critical weak point in pipeline structures, circumferential welds inevitably develop various defects during the welding process. These defects not only compromise the structural integrity and material homogeneity of the pipeline but also pose a significant risk of crack initiation and failure under the combined effects of external loads, internal pressure, and other factors [2,3,4,5,6]. Consequently, establishing effective evaluation methods to accurately assess weld defects and ensure the service safety of X80 pipeline circumferential welds has become an urgent technical requirement for pipeline engineering construction, both currently and for the foreseeable future.

Numerous standards within the oil and gas pipeline sector recommend employing fracture assessment diagrams to evaluate the safety of cracked circumferential welds. This methodology, grounded in fracture mechanics theory, integrates key parameters such as stress intensity factors and material fracture toughness. It utilises the spatial relationship between fracture assessment curves and assessment points to determine whether cracked structures meet the safety criteria. Cravero et al. [7] employed the failure assessment chart method to predict burst pressure in defective pipelines, demonstrating its feasibility through experimental data; Zhou Yawi et al. [8] employed the failure assessment diagram method to derive Charpy impact absorption values for circumferential welds applicable to the Zhongwei-Jingbian interconnection pipeline project of the West–East Gas Pipeline Phase III; Geng Liyuan et al. [9] conducted suitability assessments for pipeline circumferential welds using a hierarchical risk factor model combined with the FAD method. However, Wu Shengsi [10] contends that current failure assessment charts exhibit significant discrepancies regarding pipeline structural dimensions and load-bearing requirements compared to the design specifications and operational conditions of newly constructed high-grade steel pipelines in China. Wu [11] et al. highlighted multiple shortcomings in existing failure assessment chart methodologies, such as their inability to accurately account for strength matching characteristics of circumferential welds. Consequently, researchers have conducted extensive studies on the precision and applicability of failure assessment chart methods. Lee et al. [12] employed standard small specimens to test weld mechanical properties, constructing material-specific FAD curves that yielded more accurate assessments than generic curves. Yang Hui et al. [13] compared the principles, characteristics, and applicability of API 1104, GB/T 19624, SY/T 6477, BS 7910, and API 579 for assessing planar defects in circumferential welds. They ultimately recommended BS 7910 due to its more detailed specifications concerning residual stress selection and weld strength matching. Li An [12] conducted an engineering critical assessment of defective circumferential welds using BS 7910, validating the accuracy of the FAD method for defect evaluation in pipeline circumferential welds through wide-plate testing; Pisarski [14] validated the applicability of BS 7910 and API 1104 by collating international wide-plate tensile test data. Results indicated that although assessment outcomes differed, both standards met fundamental safety evaluation requirements. Compared to API 1104, Xu et al. [15] compared the failure load predictions of API 1104 and BS 7910 for a given defect size, finding BS 7910’s predictions to be more conservative.

A literature review indicates that although failure assessment charts have gained widespread application in pipeline engineering, significant controversy persists regarding the applicability and conservatism of various assessment methods. Currently, although wide-plate tensile tests are commonly employed to validate failure assessment chart methods, their actual stress states and defect constraint conditions differ markedly from those in service pipelines, making it difficult to accurately reflect the overall performance of ring welded joints. In light of this, this study systematically designed and conducted two sets of full-scale pressure tensile tests on large-diameter X80 pipeline ring welds, comprehensively considering key factors such as welding processes, joint configurations, defect dimensions, and locations. Concurrently, typical failure assessment chart methods were employed to evaluate pre-fabricated defects. By comparing assessment outcomes with experimental data, the applicability and conservatism of each method were thoroughly validated. The findings of this research hold significant implications for guiding engineering practice.

## 2. Common Failure Assessment Diagram Methods

Currently, there are two primary evaluation methods for failure assessment diagrams: stress-based and strain-based approaches. Although strain-based evaluation criteria are theoretically more robust, they suffer from drawbacks in practical application, including high computational complexity, substantial data requirements, and limited applicability. In contrast, stress-based evaluation criteria demonstrate clear advantages in theoretical completeness, experimental convenience, engineering flexibility, and fracture criterion effectiveness. Consequently, this paper selects the stress-based evaluation method for validation. Currently, stress-based evaluation methods commonly employed in the oil and gas pipeline sector include BS 7910 [16], API 579 [17], and API 1104 [18]. Among these, BS 7910 is widely applied for assessing various defect types, API 579 covers the majority of complex operating conditions, and API 1104 stands as one of the primary standards currently adopted in engineering practice.

### 2.1. BS 7910

BS 7910 is a standard issued by the British Standards Institution (BSI), covering the vast majority of defect types. It applies not only to welded structures but also to structures made from other metallic materials and non-welded components. The assessment methods it provides are primarily divided into three levels:

(1) Level 1 Assessment (Simple Assessment)

Level 1 assessment comprises two methods: 1A and 1B. These methods require minimal data and feature a simplified evaluation process. The 1A assessment employs failure assessment diagrams, establishing failure assessment curves for defect evaluation. The 1B assessment (also known as the equivalent crack method) does not utilise failure assessment diagrams; instead, it determines defect acceptability through defect combination analysis and tabulated calculations.

(2) Level 2 Assessment (Standard Assessment)

This level encompasses two assessment methods: 2A and 2B. Compared to Level 1 assessment, it requires more detailed data and yields more precise results.

  ① Level 2A Assessment

Level 2A assessment employs a universal assessment curve applicable to most materials, eliminating the need for stress–strain curves. Its FAD assessment curve is determined by Equation (1).

(1) $$\begin{array}{ll} K_{r}= & \left(1-0.14{L_{r}}^{2}\right)\left[0.3+0.7\exp\left(-0.65{L_{r}}^{6}\right)\right],L_{r}\leq L_{max}\\ K_{r}= & 0,L_{r}>L_{max} \end{array}$$

In the equation, *L_r_* denotes the load ratio, dimensionless; *K_r_* denotes the fracture ratio, dimensionless.

② Level 2B Assessment

The Level 2B assessment method constructs a failure evaluation curve based on the material’s true stress–strain curve, comprehensively reflecting the material’s mechanical properties. This method has a broad scope of application, covering virtually all types of base materials and welds, with significantly higher assessment accuracy than the Level 2A method. Its assessment curve is as follows:

(2) $$\begin{array}{ll} K_{r}= & {\left(\frac{E\varepsilon_{ref}}{L_{r}\sigma_{s}}+\frac{\sigma_{s}L_{r}^{3}}{2E\varepsilon_{ref}}\right)}^{-0.5},L_{r}\leq L_{max}\\ K_{r}= & 0,L_{r}>L_{max} \end{array}$$

In the equation, *σ_s_* denotes the yield strength in MPa; *E* represents the elastic modulus in GPa; *ε_ref_* corresponds to the strain at *L_r_σ_s_* on the true stress–strain curve.

(3) Level 3 Assessment (Ductile Tear Assessment)

This assessment level primarily applies to plastic materials exhibiting stable tear behaviour, whilst also being suitable for evaluating high-risk structures and complex defects. Level 3 assessment employs detailed fracture mechanics analysis methods, requiring in-depth investigation through experimentation and numerical simulation of crack propagation paths, propagation rates, and material plastic deformation behaviour. This assessment level comprises three distinct methods: 3A, 3B, and 3C, each possessing its own dedicated assessment curve. Assessment results may be presented as a single assessment point or a continuous assessment curve. When the coordinates of the assessment point or the trajectory of the assessment curve fall within the failure assessment zone, the defect-containing structure is deemed safe; conversely, failure risk exists [19].

### 2.2. API 579

API 579 is an assessment standard developed by the American Petroleum Institute (API), primarily used to evaluate the suitability of in-service pressure equipment such as pressure vessels, piping systems, and storage tanks with defects or damage, ensuring operational safety. This standard employs a failure assessment diagram-based analytical approach, dividing the assessment process into three levels.

The three-level assessment system of API 579 has distinct scopes of application and assessment accuracies:

Level 1 Assessment employs a fundamental and conservative evaluation methodology, primarily based on simplified calculations and empirical formulas. It is suitable for preliminary suitability assessments of common defect and damage types.

Level 2 provides a more detailed evaluation scheme, consistent with the generic assessment curve for Class 2A in BS 7910, as shown in Equation (1). This method enhances assessment accuracy through the introduction of more complex computational models and reasonable assumptions. It is particularly applicable when Level 1 assessment results are inconclusive or when greater precision is required.

The Level 3 assessment represents a comprehensive advanced evaluation methodology, consistent with the specific material curve for Class 2B in BS 7910, as shown in Equation (2). Utilising advanced computational techniques such as finite element analysis (FEA), this approach enables accurate evaluation of complex geometries, non-linear material properties, or defects with high uncertainty, thereby yielding optimal assessment outcomes.

### 2.3. API 1104

The API 1104 standard similarly establishes a three-tier assessment system, with increasing complexity and precision across levels. Specifically,

Level 1 assessment employs a graphical method, evaluating structural failure risk by determining critical defect sizes and comparing them with actual engineering defect dimensions.

Level 2 assessment utilises the failure assessment diagram (FAD) method, whose assessment curves are identical to those in the BS 7910 standard’s Level 2A assessment.

Level 3 assessment primarily addresses the evaluation of welded structures under cyclic loading (applicable only where fatigue spectrum severity exceeds 5 × 10^6^ cycles). This method employs the permissible defect dimensions from Level 1 assessment as the initial crack sizes for buried and surface defects. Combined with fatigue analysis per BS 7910, it establishes defect acceptance criteria.

## 3. Full-Scale Pressure-Tension Test of X80 Pipe Ring Welded Joints

**Figure 1:**
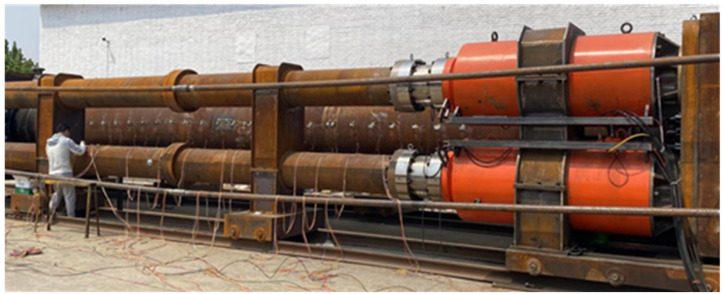
120 MN full-scale pipe tensile testing platform.

In June 2023, the National Engineering Research Centre for Oil and Gas Pipeline Transportation Safety successfully established a 120 MN full-scale pipeline tensile testing platform (Figure 1). Capable of applying loads up to 12,000 tonnes, this platform facilitated full-scale pressure tensile tests on defective X80 ring welded joints. The chemical composition of the test pipelines is detailed in Table 1.

### 3.1. Full-Scale Pressure Tensile Test on X80 Ring Welded Joints with Conventional Routes Containing Defects

Considering both automatic welding and combined automatic welding methods, root weld fusion line defects and cover weld centre defects in two positions, along with three defect dimensions (a = 3 mm/2c = 50 mm, a = 3 mm/2c = 100 mm, a = 6 mm/2c = 100 mm) where a denotes crack depth and 2c denotes crack length, the design involves conducting full-scale pressure-loaded tensile tests on standard X80 ring welded joints. Full-scale pressure-tension tests were conducted on X80 ring welded joints for conventional pipelines.

**Figure 2:**
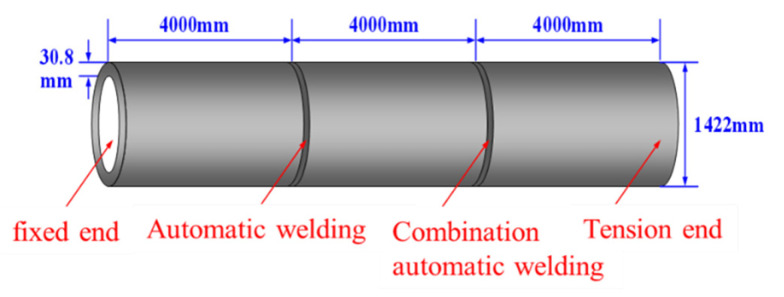
Schematic diagram of full-scale pressure tensile test for X80 pipeline ring welded joints in conventional routes.

**Figure 3:**
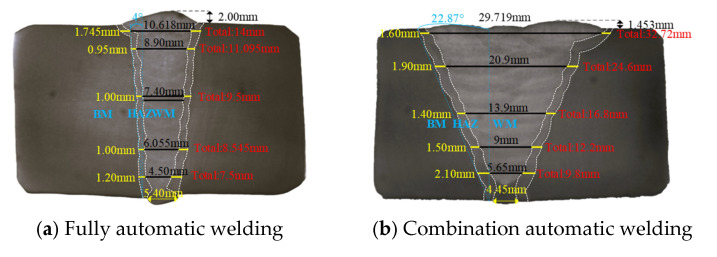
Microstructural morphology of two welding processes for ring joints.

**Figure 4:**
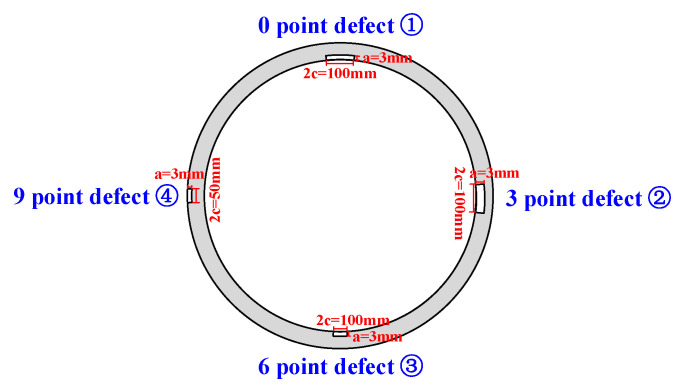
Schematic diagram of defect processing locations.

The pipeline steel grade was X80, with the total test pipe length measuring 12.0 m. The pipe diameter D was 1422.0 mm and wall thickness t was 30.8 mm. Two welding processes were employed: GMAW (Gas Metal Arc Welding) fully automated welding near the testing machine’s fixed end, and FCAW-G (Gas-Cored Arc Welding) combined automated welding near the tensile end. A schematic of the test pipeline is shown in Figure 2, with metallographic sections of both welded joints depicted in Figure 3. Mechanical machining was employed to create circumferential linear grooves at four positions on both the fully automated weld and the combined automated weld ring joints, as illustrated in Figure 4. The 0 o’clock, 3 o’clock and 6 o’clock positions all represent defects within the root weld fusion line, while the 9 o’clock position denotes a defect outside the centre of the cover weld. Details such as defect dimensions are shown in Table 2.

**Figure 5:**
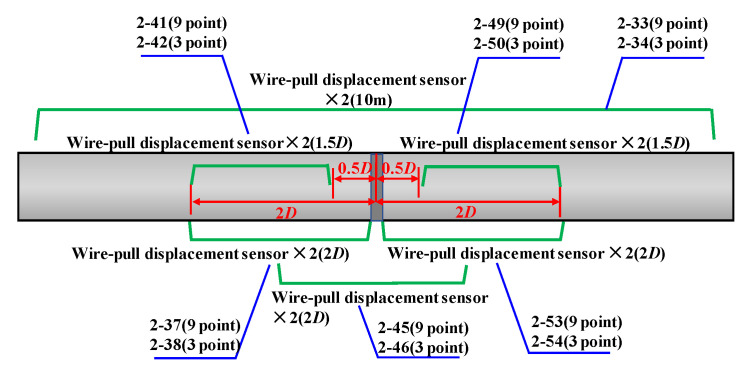
Installation layout of wire displacement sensors.

**Figure 6:**
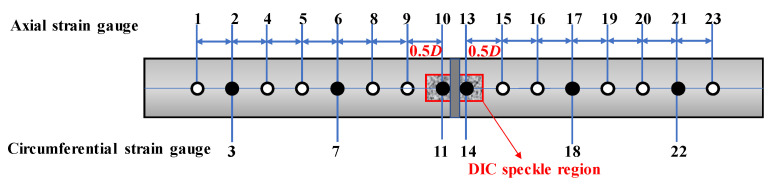
Installation layout of strain gauges.

Data acquisition employed wire-drawing displacement sensors, strain gauges, and a DIC (Digital Image Correlation) system. The wire-drawing displacement sensors were arranged as shown in Figure 5, all positioned parallel to the pipe’s axial direction and spanning the full-scale pipe length of 10.0 m, with a 2D span across the weld seam, and 1.5D and 2D spans, respectively, on either side of the weld. All sensors at identical axial positions were installed at both the 3 o’clock and 9 o’clock circumferential locations. A total of 16 strain gauges were deployed as shown in Figure 6: a total of 14 were symmetrically arranged along both sides of the circumferential weld on the base metal, while 2 were positioned on either side of the circumferential weld, both at the 3 o’clock location. Hollow circles denote axial strain gauges, while solid circles represent circumferential plus axial strain gauges. The DIC speckle projection area is a rectangle centred on the weld’s 3 o’clock position, measuring 220 mm × 410 mm, as illustrated in Figure 6.

During the test procedure, internal pressure was first applied to the pipeline. Water was pressurised to 0.5 MPa and maintained at this pressure for 10 min to verify the absence of leakage. Concurrently, all monitoring instruments were activated to confirm their operational integrity. Subsequently, pressure was increased at a rate of 1 MPa/min to 12 MPa, where it was held constant. Following completion of the internal pressure application, axial loading commenced. This was performed using a hydraulic cylinder operating in displacement control mode, with a displacement rate of 1 mm/min. The test recorded a maximum load of 71,651.2 kN and a maximum axial stress of 671.0 MPa. At the conclusion of the test, cracking occurred only at three points on the combined automatic welding head.

**Figure 7:**
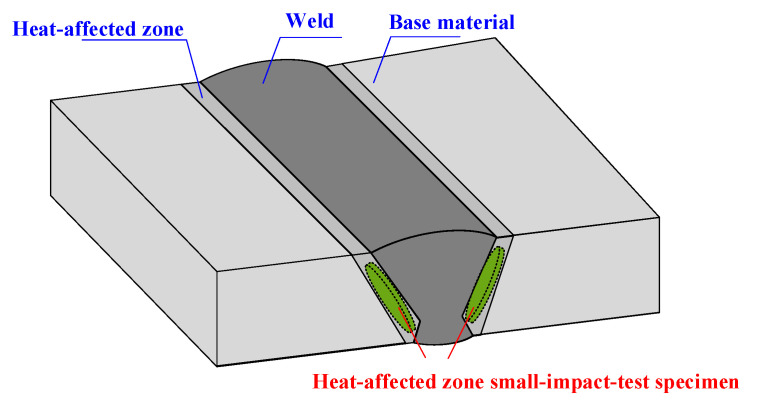
Schematic diagram of sampling for small punch specimens from the heat-affected zone of welded joints.

**Figure 8:**
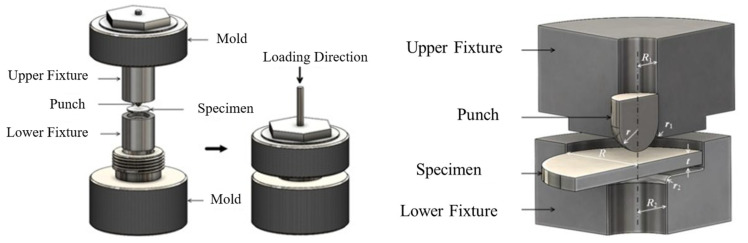
Small punch rod test clamping fixture.

**Figure 9:**
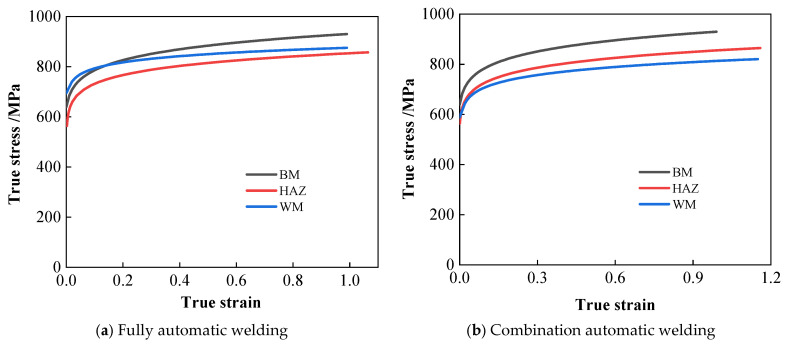
True stress–strain curve for the welded joint of the conventional X80 ring pipeline.

In accordance with the standard, uniaxial tensile tests and Charpy impact tests were conducted on both welds, yielding stress–strain curves and Charpy impact values (CVN) for various locations within the welds. Given the limited area of the heat-affected zone, standard testing methods proved inadequate for accurately characterising its mechanical properties. Consequently, the small-impact-bar test method [20,21] was employed to assess these properties. First, the welded joint specimens underwent preliminary grinding, polishing, and acid etching until the base metal zone, heat-affected zone, weld zone, and all weld passes were clearly visible. Subsequently, standard specimens were extracted according to the sampling method illustrated in Figure 7, ensuring specimens were entirely derived from the heat-affected zone. Subsequently, the extracted specimens undergo grinding to eliminate work-hardened zones resulting from the electrical discharge machining process, while further ensuring surface parallelism and roughness. Both surfaces of the specimen are ground in different directions using sandpaper until a standard-sized small punch specimen with a thickness of t = 0.500 mm (±0.005 mm) is obtained. The specimen is then clamped in an SPT-10 small punch testing machine for testing. The machine and test fixture are shown in Figure 8. The small punch test yields a material load–displacement curve. Combined with empirical correlation rules, this enables the derivation of strength performance parameters for the heat-affected zone and stress–strain curves. The material performance parameters measured at various locations for the fully automatic welding head and the combined automatic welding head are presented in Table 3. The true stress–strain curves for the material are illustrated in Figure 9.

### 3.2. Full-Scale Pressure Tensile Testing of Unequal-Wall-Thickness X80 Ring Welded Joints

Due to variations in design coefficients and wall thicknesses along pipeline routes, welding of unequal-wall-thickness steel pipes is common in field construction, particularly in complex mountainous terrain. Consequently, a series of full-scale pressure tensile tests on unequal-wall-thickness X80 ring welded joints was designed to investigate the applicability of current failure assessment chart methodologies.

**Figure 10:**
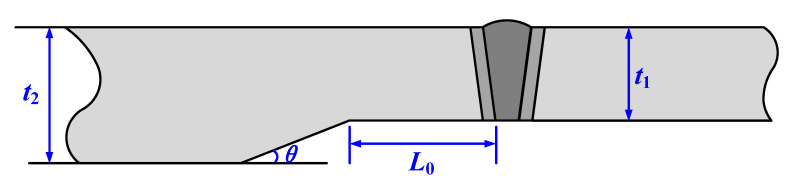
Geometric groove forms for tapered welded joints.

**Figure 11:**
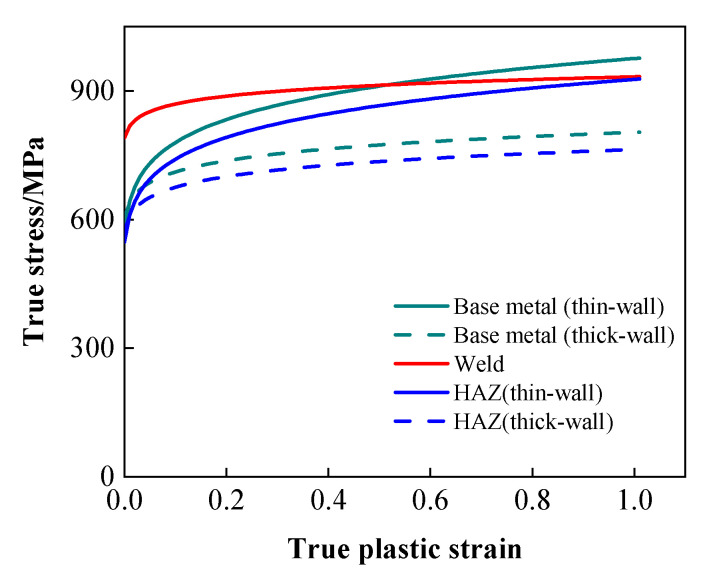
True stress–strain curve for a ring welded joint connecting X80 pipes with unequal wall thicknesses.

Pipeline specifications comprised D1422 mm × 30.8 mm and D1422 mm × 38.5 mm. Ring welds were executed via fully automated GMAW welding. An oblique straight-line hole-cone internal transition segment facilitated the transition between the two unequal-wall-thickness pipes. Geometric parameters of the hole-cone welded joint are illustrated in Figure 10. In the figure, t_1_ and t_2_ denote the wall thicknesses of the thin-walled and thick-walled sides, respectively. θ represents the transition cone angle employed in the hole-cone-shaped internal transition section, set at 15°. L_0_ denotes the transition length of the hole-cone-shaped weld joint’s internal transition section, set at 130 mm. A circumferential linear defect with a processing length of 2c (50 mm) and depth a (3 mm) was machined along the root fusion line at position 3, adjacent to the thin-walled pipe. The loading procedure followed that of conventional full-scale pressure tensile tests for X80 ring welded joints. Unloading commenced when the load reached 77,935.54 kN. The mechanical properties of the base metal and welded joint regions were obtained using the same methodology as previously described, as shown in Table 4 and Figure 11, respectively. No cracking occurred in the pre-fabricated defect at the conclusion of the test.

## 4. Validation of the Applicability of Failure Assessment Diagram Methodology

**Figure 12:**
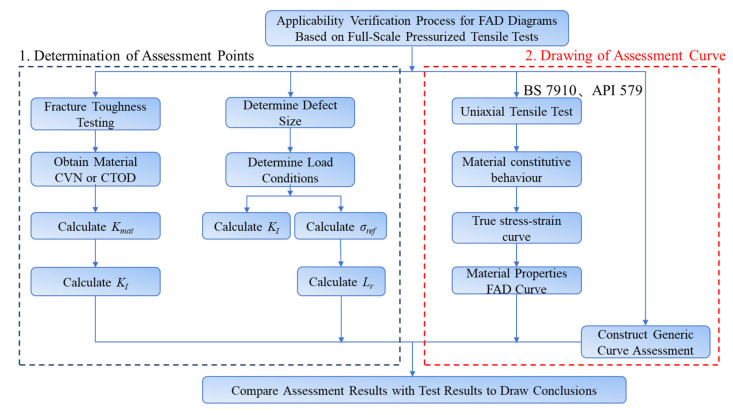
Validation process for FAD applicability based on full-scale testing.

Based on the nine sets of data obtained from the two groups of full-scale pressure-tension tests conducted on X80 pipe ring welds, the applicability of the three commonly used failure assessment chart methods introduced in Part Two—namely BS 7910, API 579, and API 1104—was verified. The specific procedure is illustrated in Figure 12.

### 4.1. Determination of Evaluation Points

(1) Reference Stress

For full-scale pressure tensile tests, the pipe is subjected to both internal pressure and tensile loading. Concurrently, due to the non-perfect symmetry of the defect, it induces certain bending stresses. Therefore, a reference stress calculation model for circumferential surface defects under the coupled effects of tensile, bending, and internal pressure is employed. Table 5 provides the reference stress calculation formulas from BS 7910, API 579, and API 1104, respectively.

In the equation, Pm denotes the principal stress, MPa; Pb denotes the bending stress, MPa; α denotes the ratio of the pipe’s internal to external diameter, dimensionless; a denotes the defect depth, mm; c denotes the defect half-length, mm; Z denotes the calculation factor, dimensionless.

(2) Fracture toughness

A key parameter influencing defect assessment is fracture toughness *K_mat_*, which denotes a material’s resistance to fracture failure in the presence of a crack. For high-toughness steels, *K_mat_* can be derived from the Charpy impact test results by converting to *CVN*s. Table 6 provides the CVN-based *K_mat_* conversion formulas specified in BS 7910, API 579, and API 1104, respectively.

In the formula, CV denotes Charpy impact energy, J; t represents the pipe wall thickness, mm; Y/T signifies the yield-to-tensile strength ratio.

(3) Stress Intensity Factor

The stress intensity factor *K_I_* is a function of working stress, crack size, and crack geometry. It is commonly employed to characterise the intensity of the stress field at the crack tip and serves as a crucial parameter for assessing structural failure. The calculation formulas for the stress intensity factor as specified in various codes are presented in Table 7.

In the formula, M_m_ and M_b_ denote the stress intensity factor multipliers for axial and bending loads, respectively, in structures with surface cracks; P_m_ represents the principal stress in MPa; P_b_ denotes the bending stress in MPa; G_0_ and G_1_ are stress intensity influence factors obtainable from tables; detailed calculation formulas for σ_0_, σ_1_, and pc are provided in Section 9.4 of API 579. It is evident that the API 579 method imposes stringent requirements on foundational data and involves a complex calculation process, whereas the BS 7910 method is comparatively straightforward to implement.

(4) Load Ratio and Fracture Ratio

The load ratio Lr characterises the extent of plastic failure, determined by the primary loads acting upon the pipeline and circumferential weld. Its calculation formula is

(3) $$L_{r}=\frac{\sigma_{ref}}{\sigma_{f}}$$

The fracture ratio Kr characterises the degree of ductile fracture, expressed as the ratio of the stress intensity factor to fracture toughness. The calculation formulae for BS 7910 and API 579 are given by Equation (4), while that for API 1104 is provided by Equation (5).

(4) $$K_{r}=\frac{K_{I}}{K_{mat}}$$

(5) $$K_{r}=\sqrt{\frac{\delta_{e}}{\delta_{mat}}}$$

The load ratio and fracture ratio of defects in two full-scale pressure-tension tests were calculated using the 2B assessment from BS 7910 and the Level 2 assessment from API 579 and API 1104, respectively. The results are presented in Table 8. Material property parameters at the defect location were employed throughout the calculations.

### 4.2. Failure Assessment Diagram Construction

The BS 7910 Grade 2B assessment and API 579 Grade 2 assessment employ identical material-specific evaluation methodologies: Select Lr values of 0.7, 0.9, 0.98, 1.0, 1.02 up to Lr_max_. Calculate the corresponding stress values using σ = Lr_σs_, obtain the corresponding ε_ref_ values from the material’s true stress–strain curve, then compute K_r_ values using Equation (2) to plot the specific material assessment curve; in contrast, the API 1104 Level 2 assessment employs a universal curve evaluation method, requiring only the substitution of characteristic points into Equation (1) to complete the assessment curve. By substituting the assessment points from Table 8 into the corresponding evaluation curve and analysing the spatial relationship between the failure assessment curve and the assessment points, the safety of the pre-fabrication defect can be determined.

**Figure 13:**
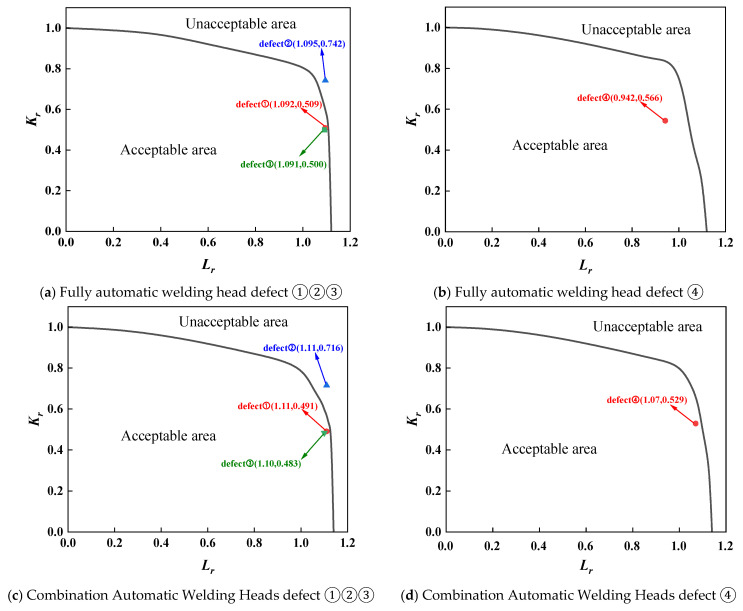
Full-scale pressure pull test results for conventional X80 pipeline ring welded joints under pressure, assessed to BS 7910.

**Figure 14:**
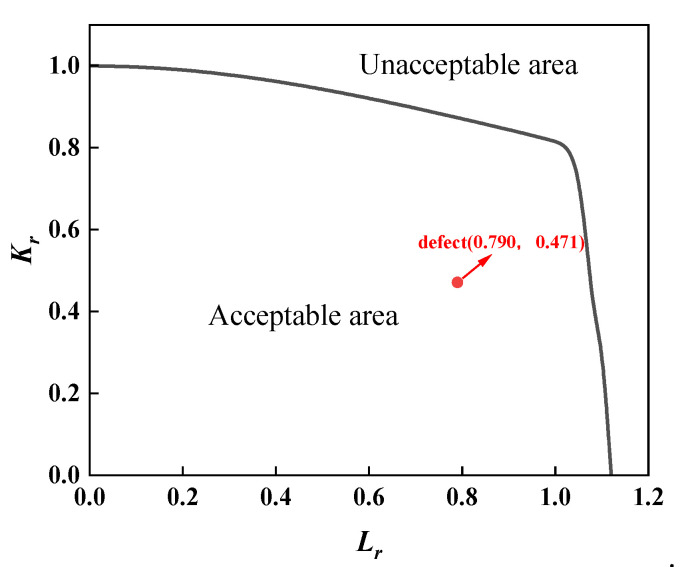
Full-scale pressure-tension test results for BS 7910 evaluation of X80 ring welded joints with unequal wall thicknesses.

**Figure 15:**
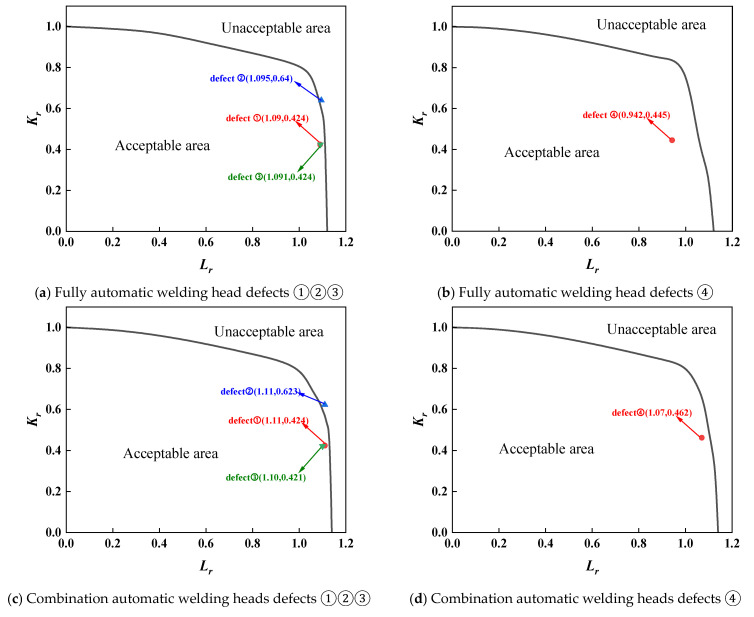
Full-scale pressure-tension test results for API 579 evaluation of standard X80 ring welds.

**Figure 16:**
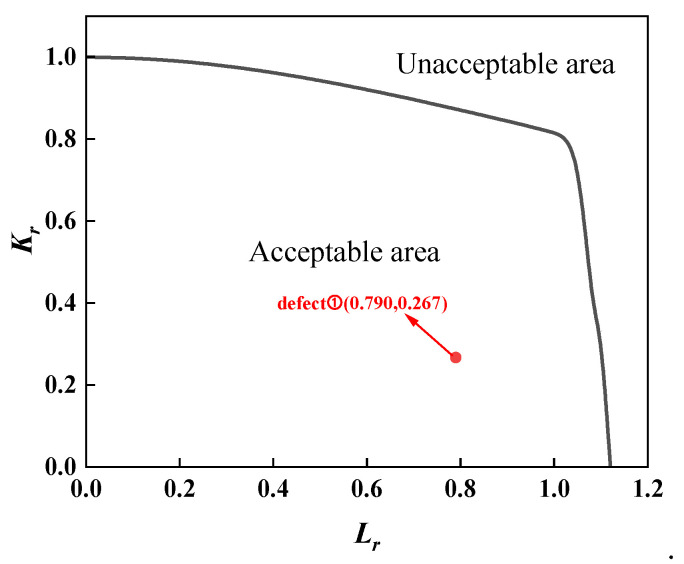
Full-scale pressure-tension test results for X80 ring welded joints with unequal wall thicknesses: API 579 evaluation.

**Figure 17:**
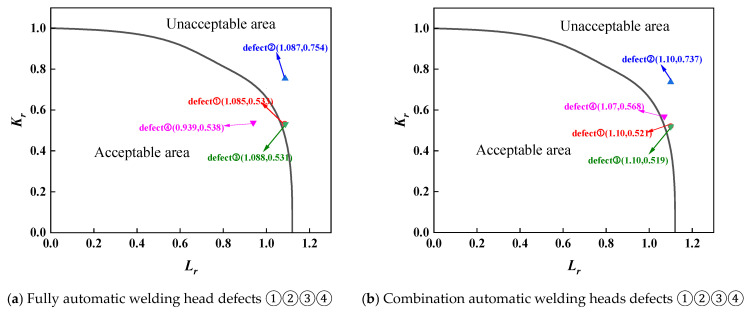
Full-scale pressure-tension test results for conventional X80 ring welded joints under pressure, evaluated to API 1104.

**Figure 18:**
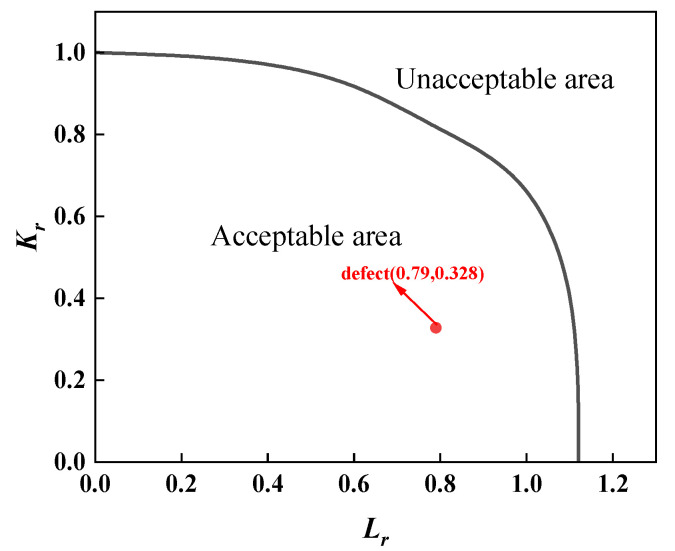
Full-scale pressure-tension test results for X80 ring welded joints with unequal wall thicknesses, evaluated per API 1104.

For the assessment of the first group of full-scale pressure-tension tests on X80 pipeline ring welds from conventional pipelines, separate evaluations were required due to material property differences between fully automatic welds and combination automatic welds. Furthermore, as defects at positions 0, 3, and 6 were root fusion line defects, while the defect at position 9 was a centre cover weld defect, material properties also differed, necessitating separate assessments. The assessment results for both sets of full-scale pressure-tension tests were analysed using three standards: BS 7910 results are shown in Figure 13 and Figure 14; API 579 results in Figure 15 and Figure 16; and API 1104 results in Figure 17 and Figure 18.

A comparative analysis of the calculated results from each assessment criterion against the test results is detailed in Table 9. The analysis indicates that, across nine defect assessment groups, both BS 7910 and API 579 yielded matching results with the test outcomes in eight groups. Only in the assessment of a three-point defect in a fully automated welded joint on a conventional pipeline did both standards predict failure, whereas no cracking or plastic deformation failure was observed in the actual test. This indicates that BS 7910 and API 579 provide generally reliable weld defect assessments, albeit slightly conservative in isolated instances. Conversely, API 1104 yielded inconsistent results with experimental data in seven out of nine defect groups, with all assessments predicting failure. This demonstrates that API 1104 adopts an overly conservative approach to weld defect evaluation, potentially leading to unnecessary repairs of otherwise qualified welded joints.

**Figure 19:**
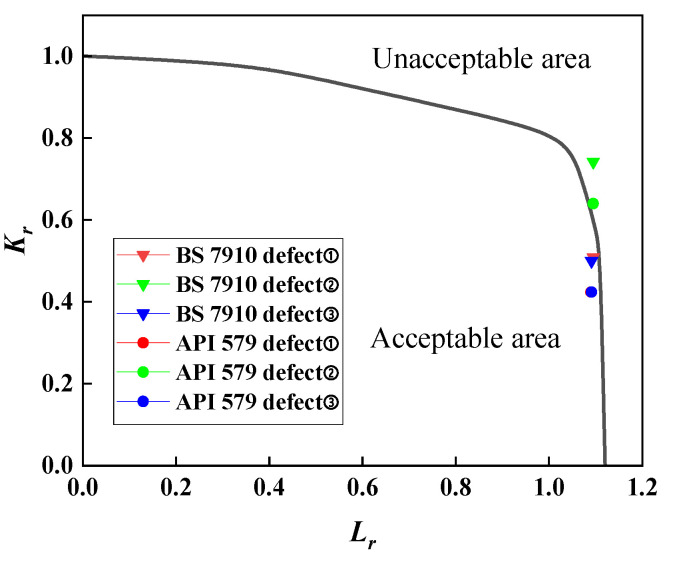
Comparison of API 579 and BS 7910.

To further compare the conservatism of BS 7910 and API 579, considering both employ specific material curves with identical failure assessment curves, defects ①②③ from a conventional pipeline fully automatic welding head were selected as examples. Their assessment point coordinates were substituted into the same assessment diagram for comparison, as shown in Figure 19. Comparing the coordinates of the assessment points between BS 7910 and API 579 reveals that, for similar horizontal coordinate values, the vertical coordinate values for BS 7910 are higher. This indicates a greater crack propagation driving force, suggesting a higher risk of fracture failure. Furthermore, the assessment points are positioned closer to the right-hand side of the coordinate axes compared to API 579, i.e., nearer to the unacceptable zone. This indicates that BS 7910 yields more conservative assessment results than API 579.

In summary, when assessing defects in ring welds similar to those tested under these conditions (high-strength grade X80, large diameter, ring welds with specific welding procedures), the BS 7910 standard offers a choice that combines good precision with safety-oriented conservatism. Its graded assessment method provides researchers with flexibility to adjust the level of conservatism according to the evaluation objectives. The data from this study provides valuable experimental reference for selecting assessment methods under specific conditions (such as similar materials, defects, and loading patterns). It must be emphasised, however, that the conclusions drawn from this study are based on a specific range of tests, whereas the selection of engineering standards requires consideration of multiple factors.

## 5. Conclusions

(1) Full-scale pressure-bearing tensile tests were conducted on conventional and unequal-wall-thickness pipeline ring welds. Utilising a multi-source data acquisition system, test data was successfully obtained, enabling the safety assessment of pre-fabrication defects in pipeline ring welds under actual operating conditions within a biaxial stress state.
(2) Comparative analysis of full-scale pressure-tension test results against failure assessment diagram methodology indicates that for high-grade steel, large-diameter, thick-walled pipe ring welds, the API 1104 method yields lower assessment accuracy due to excessive conservatism. The predictive accuracy of both API 579 and BS 7910 methods is comparable, with both capable of adequately assessing defect safety. However, BS 7910 exhibits greater conservatism, offering enhanced safety assurance from an engineering application perspective.
(3) Under the specific material and defect assessment employed in this study, the evaluation results of API 1104 demonstrated significant conservatism (two sets in agreement) under the research conditions, whereas the assessment outcomes of BS 7910 and API 579 showed higher concordance with the test results (eight sets in agreement). BS 7910’s assessment points demonstrate a relatively higher safety margin compared to API 579 under conditions involving high-grade steel large-diameter pipelines.

## Figures and Tables

**Table 1:** Chemical composition of X80 steel pipes.

Pipeline Steel Grades	C /%	Si /%	Mn /%	P /%	S /%	Cr /%
X80	0.043	0.23	1.87	0.01	0.003	0.025
Mo /%	Ni /%	Nb /%	V /%	Ti /%	Cu /%	Fe /%
0.27	0.23	0.06	0.006	0.017	0.13	Bal.

**Table 2:** Weld defect pre-fabrication conditions.

Serial Number	Circumferential Weld Seam Location	Location	Defect Dimensions
①	0 point	root weld fusion line	*a* = 3 mm/2*c* = 100 mm
②	3 point	root weld fusion line	*a* = 6 mm/2*c* = 100 mm
③	6 point	root weld fusion line	*a* = 3 mm/2*c* = 50 mm
④	9 point	Cover Welding Centre	*a* = 3 mm/2*c* = 50 mm

**Table 3:** Material properties of conventional X80 ring welded joints.

Welding Method	Sampling Location	*σ*_Y0.5_/MPa	*σ*_U_/MPa	CVN/J
Fully automatic welding	base material	620	710	252.3
	Heat-affected zone	564	668	176.0
	weld	695	733	137.4
Combination automatic welding	base material	620	710	252.3
	Heat-affected zone	551	662	189.0
	weld	589	648	145.2

In the table, σ_Y0.5_ denotes yield strength in MPa; σ_U_ denotes tensile strength in MPa; CVN denotes Charpy impact energy in joules.

**Table 4:** Material properties of X80 ring welded joints with unequal wall thicknesses.

Sampling Area	Sampling Location	*σ*_Y0.5_/MPa	*σ*_U_/MPa	CVN/J
base material	Thin-walled pipe side	576.5	701	250.75
	Thick-walled pipe side	603	654	354.35
Heat-affected zone	Thin-walled pipe side	548	666	270
	Thick-walled pipe side	573	621	325.83
weld	/	790.5	820	206

**Table 5:** Reference stress calculation formulas for various specifications.

Standard	Formula for Calculation
BS 7910	$\sigma_{ref}=\frac{p_{m}[\pi (1-a/t)]+2a/t\sin(a/t)}{\left(1-a/t)[\pi -(c/r_{i})(a/t)\right]}$
API 579	$\sigma_{ref}=\frac{P_{b}+{\left(P_{b}^{2}+9{\left\{Z\cdot P_{m}\cdot {\left(1-\alpha \right)}^{2}\right\}}^{2}\right)}^{0.5}}{3{\left(1-\alpha \right)}^{2}}$
API 1104	$\sigma_{\mathrm{ref}}=\left[\frac{\pi }{4}+385{\left(0.05-2ac\right)}^{2.5}\right]\left[\cos\left(ac\pi \right)-\frac{a\sin(2c\pi )}{2}\right]P_{m}$

**Table 6:** Formulae for calculating fracture toughness according to various standards.

Standard	Formula for Calculation
BS 7910	$K_{mat}=\left(12\sqrt{C_{V}}-20\right){\left(\frac{25}{t}\right)}^{0.25}+20$
API 579	$K_{\mathrm{mat}}=\sigma_{s}{\left[0.52\left(\frac{\mathrm{CVN}}{\sigma_{s}}-0.02\right)\right]}^{0.5}$
API 1104	$\delta_{mat}=\left(0.0086*Y/T-0.0021\right)*C_{V}$

**Table 7:** Calculation formulas for stress intensity factors in various specifications.

Standard	Formula for Calculation
BS 7910	$K_{I}=(M_{m}P_{m}+M_{b}P_{b})\sqrt{\pi a}$
API 579	$K_{I}=\left[G_{o}\left\{\sigma_{o}+p_{c}\right\}+G_{1}\sigma_{1}\left(\frac{a}{t}\right)\right]\sqrt{\pi a}$
API 1104	$K_{I}=P_{m}\sqrt{\pi a}F_{b}$

**Table 8:** Summary of calculation results for each evaluation criterion.

Test Name	Welding Method	Defect Number	BS 7910	API 579	API 1104
**Full-scale pressure-tension test on welded joints for conventional pipeline loops**	Fully automatic welding	①	(1.09, 0.509)	(1.09, 0.424)	(1.085, 0.533)
		②	(1.095, 0.742)	(1.095, 0.640)	(1.087, 0.754)
		③	(1.091, 0.500)	(1.091, 0.424)	(1.088, 0.531)
		④	(0.942, 0.566)	(0.942, 0.445)	(0.939, 0.538)
	Combination automatic welding	①	(1.11, 0.491)	(1.11, 0.424)	(1.10, 0.521)
		②	(1.11, 0.716)	(1.11, 0.623)	(1.10, 0.737)
		③	(1.10, 0.483)	(1.10, 0.421)	(1.10, 0.519)
		④	(1.07, 0.529)	(1.07, 0.462)	(1.07, 0.568)
**Full-scale pressure-loaded tensile test of welded joints with unequal-wall-thickness connecting rings**	Fully automatic welding		(0.790, 0.471)	(0.790, 0.267)	(0.790, 0.328)

**Table 9:** Comparison of full-scale pressure tensile test results with failure assessment diagram outcomes.

Test Type	Defect Number	Test Result	Consistency of Assessment Result with Test Result		
			BS 7910	API 579	API 1104
**Fully automatic welding head for conventional routes**	①	Uncracked	correspond	correspond	incongruous
	②	Uncracked	incongruous	incongruous	incongruous
	③	Uncracked	correspond	correspond	incongruous
	④	Uncracked	correspond	correspond	correspond
**Automatic welding head for standard circuit combinations**	①	Uncracked	correspond	correspond	incongruous
	②	cracked	correspond	correspond	correspond
	③	Uncracked	correspond	correspond	incongruous
	④	Uncracked	correspond	correspond	incongruous
**Welded joint for connecting rings with unequal wall thicknesses**	The only defects	Uncracked	correspond	correspond	incongruous

## Data Availability

The original contributions presented in this study are included in the article. Further inquiries can be directed to the corresponding author.
